# Isolated Langerhans cell histiocytosis of the stomach in adults: An analysis of clinicopathologic characteristics and molecular genetics

**DOI:** 10.1097/MD.0000000000040950

**Published:** 2024-12-20

**Authors:** Ruinuan Wu, Yali Zhao, Xikang Wu, Huihui Gui, Xia Liu, Zhaohui Liu

**Affiliations:** a Department of Pathology, Shenzhen Second People’s Hospital, First Affiliated Hospital of Shenzhen University Health Science Center, Shenzhen, China; b Department of Pathology, Shenzhen Longgang Central Hospital, Shenzhen, China; c Department of Gastroenterology, Shenzhen Second People’s Hospital, First Affiliated Hospital of Shenzhen University Health Science Center, Shenzhen, China.

**Keywords:** adults, isolated Langerhans cell histiocytosis, stomach

## Abstract

Isolated gastric Langerhans cell histiocytosis (LCH) occurs extremely rarely in adults. We characterized the clinicopathological and molecular genetics of this rare entity. We retrospectively analyzed the clinicopathologic and prognostic features of 3 patients with isolated gastric LCH during the past 10 years, with a review of an additional 20 patients from the literature. A total of 23 patients with isolated gastric LCH were included in this study. There were 15 males and 8 females, with a mean age of 44.5 (median, 48; range, 21–68) years. Stomach discomfort and abdominal pain were the most common presenting symptoms. The lesions were mainly concentrated in the gastric body and antrum (21/23). Gastroscopy often revealed an elevated lesion/polyp. Molecular tests showed that *BRAF-V600E* gene mutations were found in 10/11 (42%) patients, while none of the patients (0/5) harbored *KRAS* gene mutations. None of the 23 patients received further treatment. Twenty patients had follow-up results (from 4 to 66 months). One patient with atypical morphological features died of unknown cause 2 months after removal of the tumor. One patient was found to have secondary lesions in the skull and axillary region. The other 18 patients survived without any evidence of disease progression during the follow-up period. In the daily diagnosis of gastroscopic biopsy, it is necessary to be aware of the possibility of LCH in patients with lesions in the gastric body or antrum if endoscopy reveals bulge/polypoid changes and heavy microscopic inflammation. In addition, we should be alert to the possibility of LCH with malignant transformation if the histological morphology exhibits tumor cell nucleoli and mitotic figures or necrosis. The immunohistochemical marker CD56 may help differentiate between LCH and Langerhans cell sarcoma when the morphology is difficult to determine. Molecular detection has shown that the mutation rate of *BRAF* in gastric LCH is up to 90.9%; more work is needed as the number of cases is small. Current data show a good prognosis for isolated gastric LCH in adults, but long-term follow-up for early detection of disease progression or systemic involvement is necessary.

## 1. Introduction

Langerhans cell histiocytosis (LCH) is an inflammatory myeloid neoplasia in involved tissues characterized by the accumulation of abnormal cells believed to be of monocyte/macrophage lineage that harbor pathogenic mutations activating the mitogen-activated protein kinase (MAPK) pathway.^[1]^ LCH can occur at any age, mainly involving children and adolescents, with an annual incidence of 5 to 9 cases per 1,000,000 people for children younger than 15 years and at least 0.07 cases per 1,000,000 people for patients older than 18 years.^[2]^ Single-system LCH most commonly affects the bone, skin, or lymph nodes. Isolated gastric LCH in adults is an exceedingly rare disease. To date, only approximately 20 cases have been reported, mainly as single-case reports rather than as study series. Due to its rarity, more studies of isolated gastric LCH in adults are needed to better understand its clinicopathological characteristics and molecular genetics. In an attempt to improve our understanding of this rare entity, we retrospectively analyzed 3 cases of isolated gastric LCH in adults and comprehensively summarized the details of 20 additional cases from the literature.^[3–19]^

## 2. Materials and methods

### 2.1. Case selection

We collected a total of 3 cases of isolated gastric LCH in adults, including 2 cases from Shenzhen Second People’s Hospital (Shenzhen, China) and 1 from Shenzhen Longgang District Central Hospital (Shenzhen). The study was conducted in accordance with the 2008 revision of the Helsinki Declaration and was approved by the Ethics Committee of the Second People’s Hospital of Shenzhen Municipality. The inclusion criteria were as follows: imaging (computed tomography [CT] and type-B ultrasonic or positron emission CT [PET-CT]) showing no LCH in other organs and a gastric lesion as the initial presentation without bone, skin, lymph node, or lung manifestations at presentation. Patients with a prior diagnosis of LCH were excluded. The clinical data collected for analysis included age, sex, clinical history, tumor location, initial presentation, gastroscopy, and treatment. We also performed a comprehensive literature search for reported cases of gastric LCH in adults in PubMed using different combinations of keywords, including “gastric,” “stomach,” “Langerhans cell histiocytosis,” “eosinophilic granuloma,” “histiocytosis X,” “LCH,” and “adult.” A total of 21 adult patients with gastric LCH (confirmed by immunohistochemistry and/or electron microscopy) were retrieved from the literature and included in our review.

### 2.2. Hematoxylin and eosin and immunohistochemistry

The specimens were formalin-fixed, paraffin-embedded, and cut into 4-mm-thick sections, which were stained with hematoxylin and eosin and subjected to immunohistochemistry. Immunohistochemical staining was performed on a Leica BOND-III Fully Automated IHC & ISH Staining System (Leica Biosystems Newcastle Ltd, England) with a Bond Polymer Refine Detection Kit (Leica Biosystems; catalog no. DS9800). The clone and source of the antibodies were S-100 4C4.9 (Maixin Bio, Fuzhou, China), CD1a (010; Maixin Bio), langerin (12D6; Maixin Bio), CK (AE1/AE3; Maixin Bio), and Ki-67 (MXR002; Maixin Bio). Appropriate negative and positive controls were subjected to satisfactory staining.

### 2.3. Molecular assays for gene mutations

*BRAF-V600E* and *KRAS* mutations were detected in the formalin-fixed paraffin-embedded samples using real-time polymerase chain reaction (PCR). DNA was extracted using a commercial AmoyDx FFPE DNA Kit (Amoy Diagnostic Co Ltd, Xiamen, China; catalog no. 8.02.23501X036G) according to the manufacturer's instructions. Subsequently, DNA (15 ng) was examined for *BRAF-V600E* and *KRAS* mutations using commercial kits with a detection sensitivity of 1% mutation load (Human BRAF Gene V600E Mutation Fluorescence PCR Diagnostic Kit, Amoy, catalog no. 8.0120301X024A; AmoyDx KRAS Mutations Detection Kit, Amoy, catalog no. 8.01.25402 W006A) in an ABI 7500 real-time PCR machine (Applied Biosystems, CA). FAM signals from the mutation detection system indicated the mutation status of the sample.

## 3. Results

### 3.1. Clinical characteristics

**Figure 1.**
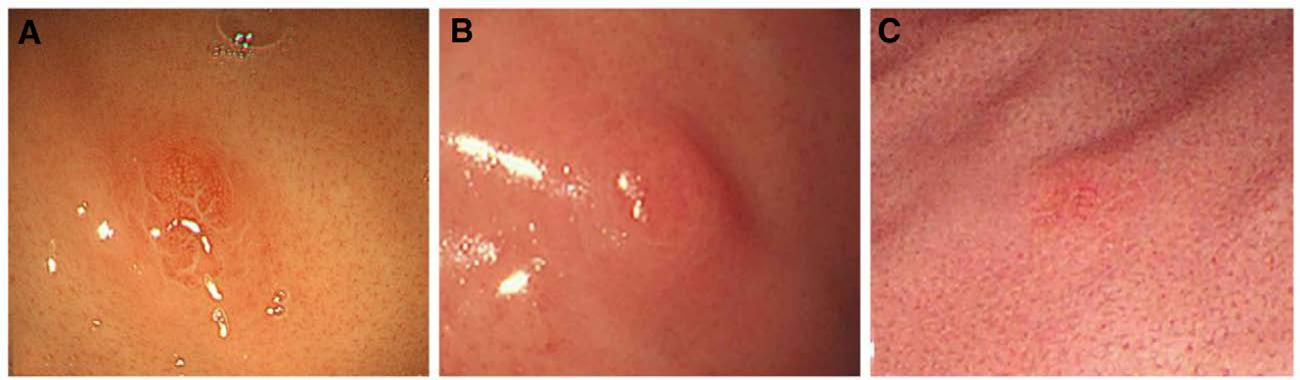
Gastroscopy of isolated Langerhans cell histiocytosis of the stomach. (A) Patient 1 showed patchy redness and a microbulge 5 × 6 mm in size in the gastric body. (B) Patient 2 showed patchy redness and a microbulge 7 × 8mm in size in the gastric body. (C) Patient 3 had a red polypoid bulge 4 mm in diameter with a smooth surface in the gastric body.

The main clinical features of our 3 patients with gastric LCH are summarized in Table 1. All 3 patients were male, with ages ranging from 21 to 40 years. None of the patients had a prior history of LCH, and thorax and skeleton computerized tomography or PET-CT of the whole body revealed no abnormalities outside the stomach at presentation. Clinically, patient 1 complained of intermittent abdominal pain for >4 months. Patient 2 complained of stomach discomfort for more than a year. Patient 3 underwent gastroscopy without any symptoms due to routine physical examination. All of the lesions were located in the gastric body. On gastroscopy, patient 1 showed patchy redness and a microbulge 5 × 6 mm in size (Fig. 1A). Patient 2 showed patchy redness and a microbulge 7 × 8 mm in size (Fig. 1B). Patient 3 had a polypoid bulge 4 mm in diameter with a smooth surface (Fig. 1C). In searching the literature, we found 20 patients with adult gastric LCH, plus 3 patients in this group, for a total of 23 patients. The clinicopathological features of the patients from the literature and our institution are summarized in Table 2. There were 15 males and 8 females, with a mean age of 44.5 (median, 48; range, 21–68) years. The most common symptoms were stomach discomfort and abdominal pain (10/23). Other presenting symptoms included upper abdominal fullness, nausea and vomiting, dysphagia, and other gastrointestinal symptoms. Nine patients had no gastrointestinal symptoms. The most common site was the stomach body (14/23), followed by the stomach antrum (5/23), the junction between the stomach body and stomach antrum (2/23), and the stomach fundus (1/23); in one patient, the lesions spread through the whole stomach (1/23). Macroscopic findings often included elevated lesions/polyps (15/23), erosion (3/23), and mucosal erythema (2/23). One patient had an ulcerated mass of 1.5 cm in diameter, one patient had a 4.5 × 2.5 cm large, spherical submucosal tumor, and one patient had normal mucosa.

**Table 1:** Clinical features of the 3 cases with gastric LCH in the present study.

Cases	Case 1	Case 2	Case 3
Age, yr/sex	21/male	32/male	40/male
Location	Gastric body	Gastric body	Gastric body
First symptoms	Abdominal pain	Stomach discomfort	Asymptomatic
Duration time, min	4	12	None
Gastroscopy	Patchy redness, microbulge	Patchy redness, microbulge	A red polypoid bulge 4 mm in diameter
Treatment	Observation	Observation	Observation
Follow-up, mo	4	5	25
Outcome (recurrence or progression/NED)	NED	NED	NED

LCH = Langerhans cell histiocytosis, NED = no evidence of disease.

**Table 2:** Clinicopathologic features of gastric LCH in the present study and the literature.

Characteristics	Present study	Literature	Total
Total cases	3	20	23
Male/female	3/0	12/8	15/8
Median age, yr	32	49	48
Location (stomach body/stomach antrum/the junction between stomach body and stomach antrum/stomach fundus/the whole stomach)	3/0/0/0/0	11/5/2/1/1	14/5/2/1/1
Macroscopic findings (elevated lesions or polyp/erosion//erythema/mass/normal mucosa)	3/0/0/0/0	12/3/2/2/1	15/3/2/2/1
BRAF mutation (positive/negative/unknown)	3/0/0	7/1/12	10/1/12
KRAS mutation (positive/negative/unknown)	0/3/0	0/2/18	0/5/18
Median follow-up, mo	0/(n = 3)	8/(n = 17)	9/(n = 20)
Outcome (recurrence or progression/NED)	0/3	2/18	2/21

BRAF = proto-oncogene, serine/threonine kinase, KRAS = Kirsten rat sarcoma viral oncogene homolog, LCH = Langerhans cell histiocytosis, NED = no evidence of disease.

### 3.2. Pathology findings

#### 3.2.1. Histology and immunophenotyping

**Figure 2.**
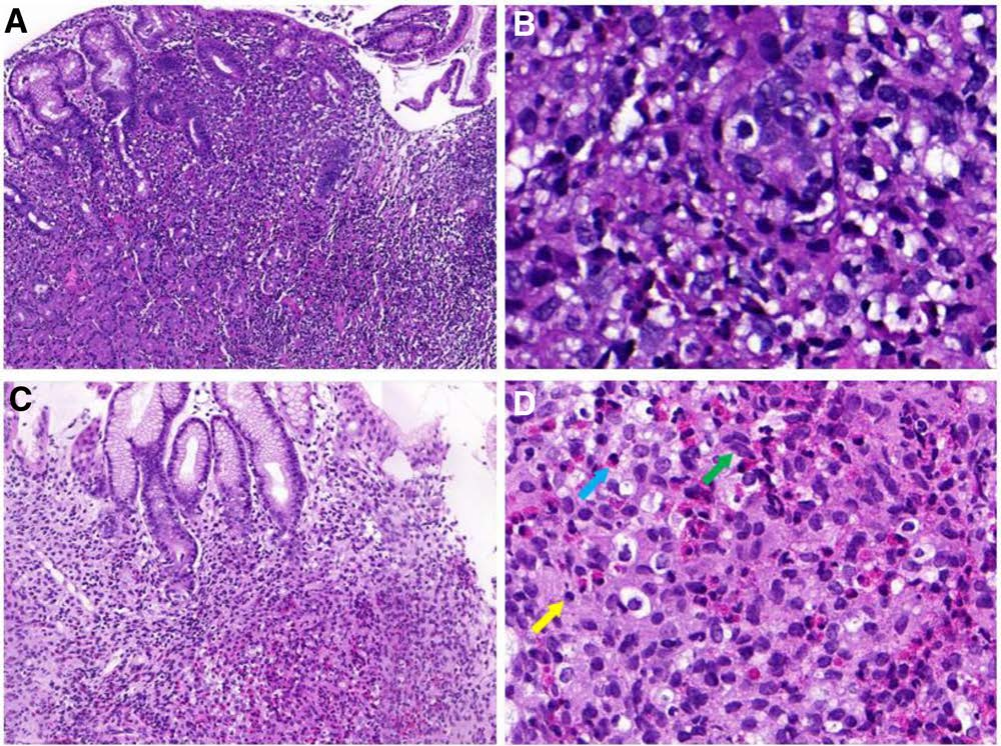
Morphology of isolated Langerhans cell histiocytosis of the stomach. (A) Langerhans cells proliferate in a patchy fashion in the lamina propria (patient 1, hematoxylin and eosin ×100). (B) clusters of Langerhans cells surrounded by the glands (patient 1, hematoxylin and eosin ×400). (C) Langerhans cells tended to form a sheet-like fashion with large numbers of eosinophil cells (patient 2, hematoxylin and eosin ×100). (D) Langerhans cells (green arrow) showing distinctive morphology of intermediate-sized cells with irregular nuclear contours, nuclear grooves and pale eosinophilic cytoplasm (patient 2, hematoxylin and eosin ×400), eosinophil (blue arrow), and lymphocyte (yellow arrow).

**Figure 3.**
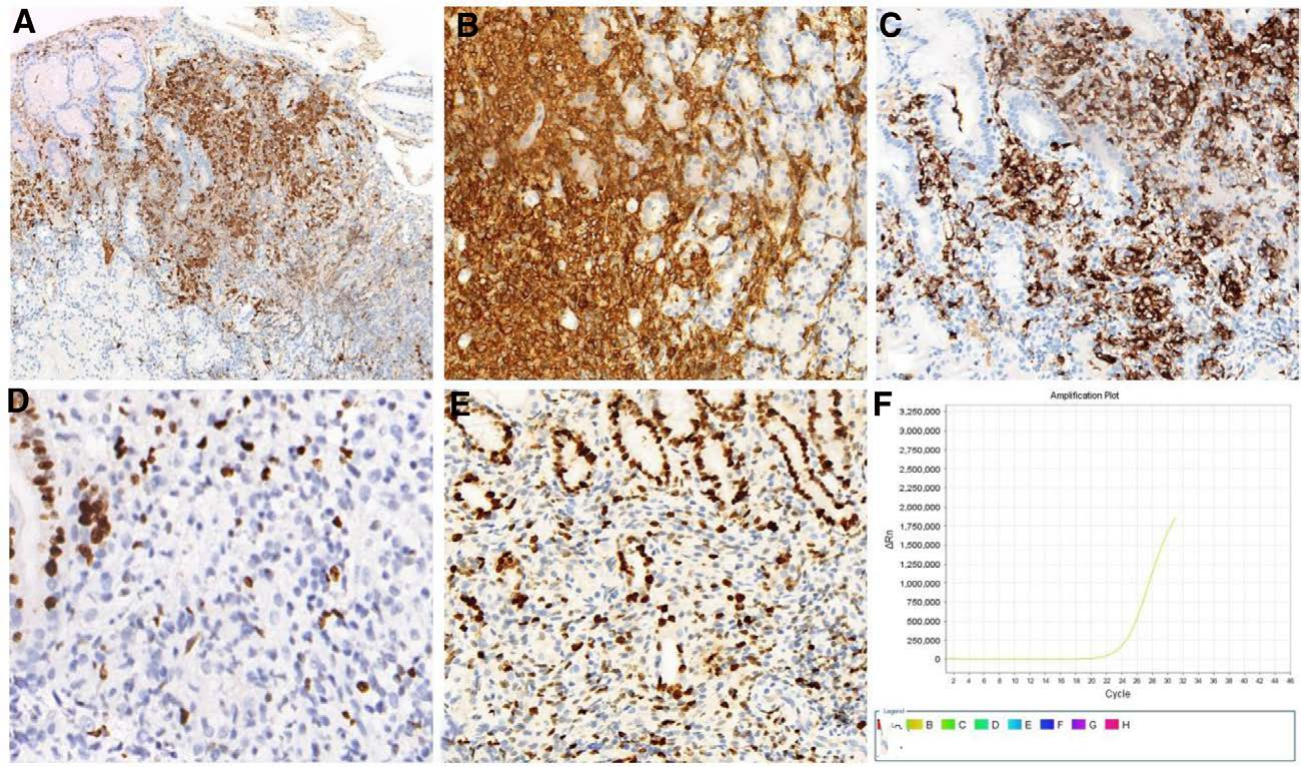
Immunophenotype and *BRAF-V600E* gene mutations of isolated Langerhans cell histiocytosis of the stomach. (A, B, and C) The large histiocytes were strongly positive for S-100, CD1a, and langerin, respectively. (D and E) The Ki-67 labeling index was 2% to 30%. (F) All patients had a *BRAF-V600E* gene mutation.

The histomorphologic features of gastric LCH are similar to those of other sites. Classically, Langerhans cells proliferate in a patchy or sheet-like fashion in the lamina propria (Fig. 2A, 2B, and 2C). The cells were round to polygonal epithelioid; the nuclei were oval to reniform; the nucleoli were not obvious, with intranuclear grooves; mitosis was rarely found; and there was pale eosinophilic cytoplasm (Fig. 2D). In addition, eosinophils and lymphocytes were mixed with these cells. Immunohistochemically, all the tumor cells were strongly positive for S-100, CD1a, and langerin (Fig. 3A, 3B, and 3C), and the Ki-67 labeling index was 2% to 30% (Fig. 3D and 3E).

#### 3.2.2. Molecular genetics

PCR assays for *BRAF-V600E* and *KRAS* gene mutations were performed on the 3 patients. All patients had a *BRAF-V600E* gene mutation but no mutations in the *KRAS* gene (Fig. 3F). Eight of the 20 patients reported in the literature underwent *BRAF-V600E* gene mutation analysis; *BRAF-V600E* mutations were found in 7 patients, and *KRAS* gene mutations were detected in 2 patients, while none of the patients harbored mutations. Combining our data and the data reported in the literature, the mutation rate of *BRAF* in gastric LCH is 10/11 (90.9%).

#### 3.2.3. Follow-up and clinical management

Patient 1 and patient 2 underwent whole-body PET-CT after diagnosis. Patient 3 underwent abdominal ultrasonography and thoracic CT after diagnosis. Other systemic involvement was excluded in these 3 patients who had isolated lesions primary to the stomach and did not receive further treatment. All 3 patients were followed up until April 20, 2024. The follow-up interval ranged from 4 to 25 months. All patients survived without any evidence of disease progression during the follow-up period.

Of the 21 patients reported in the literature, 18 patients had follow-up results with follow-up intervals ranging from 3 to 66 months. One patient was found to have secondary lesions in the skull and axillary during the follow-up period. One patient with atypical morphological features died from unknown causes 2 months after removal of the tumor.

## 4. Discussion

Clinically, LCH can be divided into unifocal, multifocal single-system, and multisystem disease. In unifocal disease, bone involvement is present in >90% of cases. One Germany-based registry reported that LCH in adult patients affects, in decreasing order of frequency, bone, skin, the pituitary gland, the liver/spleen, the brain, and the gastrointestinal tract, with the latter accounting for only 2% of cases.^[19,20]^ We identified only 20 cases of isolated LCH with primary disease in the adult stomach. Owing to its rarity, the clinicopathological characteristics and prognosis of gastric LCH have not been well described. According to the data of this group and previous reports, the male-to-female ratio was 1.875 (male: 15 and female: 8), and the lesions were mainly concentrated in the gastric body and antrum (21/23). Gastroscopy revealed a mucosal bulge/polyp (15/23). Therefore, it is necessary to be alert to the possibility of LCH when the lesion is located in the gastric body or antrum, especially in male patients, and gastroscopy reveals an elevated lesion or polyp with heavy background microscopic inflammation in the biopsy specimen.

The histological morphology of gastric LCH is similar to that of LCH in the bone. However, Luigi Terracciano reported that gastric LCH presented with an atypical morphology.^[17]^ Based on the histological morphology of typical LCH, some cells in the focal area displayed larger sizes with conspicuous nucleoli and marked cytological atypia. The mitotic index in general was low, but, in some areas, up to 4 mitoses/high-power fields and multiple areas of necrosis were found. The tumor infiltrated the gastric wall and pancreatic tissue, and the patient died of unknown causes 2 months after diagnosis. In this case, the evidence for the diagnosis of Langerhans cell sarcoma (LCS; cellular polymorphism, >50/10 high power field) was insufficient. However, if the tumor cell nucleoli are evident and mitosis can be observed, especially atypical mitosis, even though atypical cells exist only in the local visual field, we should be alert to the possibility of LCH with malignant transformation. Immunohistochemically, the tumor cells were strongly positive for S-100, CD1a, and langerin. Kawase et al^[21]^ indicated that the expression of CD56 could be important in the differential diagnosis of LCS and LCH and in the prediction of the outcome when the morphology is difficult to determine.

LCH is often characterized by activating mutations in the MAPK pathway via a specific cascade of RAS-RAF-MEK-ERK phosphorylation, which transfers signals from extracellular afferents into the nucleus through the cell membrane. As BRAF is a pivotal kinase in the RAS-RAF-MEK signaling pathway, the *BRAF-V600E* mutation abrogates dendritic cell (DC) migration trap DCs in tissue lesions and causes evasion of apoptosis in LCH.^[22]^ *BRAF-V600E* mutations have been reported in 38% to 57% of patients with nongastric LCH.^[23,24]^ Among the 23 patients included in our present study and reported in the literature, 11 cases had been tested for BRAF V600E mutations and 10 had shown mutations. Berres et al^[25]^ reported that the presence of *BRAF-V600E* in pathological DCs within LCH lesions was associated with a greater risk of refractory or recurrent disease. In addition, pathological MAPK pathway mutations in different differentiation stages of myeloid cells determine the outcome of children with LCH: mutations in bone marrow stem cells with self-renewal ability lead to multisystem disease, and *BRAF* gene or other kinase gene mutations can be detected in peripheral blood or bone marrow; progenitor mutations in the directional differentiation stage lead to multifocal single-system involvement; and with cell differentiation maturity and directional migration, somatic mutations in the late differentiation stage, the disease only manifests as unifocal single-system involvement.^[25]^ Since the cases we reported were all solitary in the stomach, we speculated that *BRAF* mutations occur in somatic cells in the late differentiation stage. Current research indicates that gastric LCH does not exhibit significant molecular differences from LCH in other locations, with the BRAF V600E mutation being the most commonly observed alteration.^[22]^ All 3 cases of gastric LCH in this study tested positive for the BRAF V600E mutation. However, these findings may be constrained by the small sample size. Future studies should aim to collect a larger number of cases and conduct comprehensive genetic testing to ascertain whether gastric LCH has unique molecular pathological changes or if its genetic alterations align with those found in LCH elsewhere in the body.

LCH with most single systems had a good prognosis, with 14 of 18 previous patients having follow-up results ranging from 3 months to 5.5 years and 12 having a good prognosis. In one patient, 2 years after the initial diagnosis, LCH lesions were found in the axillary, suprapubic, and skull regions.^[18]^ In the other patient, the tumor cell had infiltrated the gastric wall and surrounding pancreatic tissue; although the diagnosis at that time was LCH with atypical morphological features, this patient was more likely to experience local malignant transformation.^[17]^ Progression to LCS has also been reported in LCH of the lung.^[26]^ Patients in this group remained well with follow-up from 4 months to 25 months, but long-term follow-up for early detection of disease progression or systematic involvement is necessary.

In conclusion, isolated gastric LCH in adults is an exceedingly rare disease. In the daily diagnosis of gastroscopic biopsy, it is necessary to be aware of the possibility of LCH in patients with lesions in the gastric body or antrum if endoscopy reveals bulge/polypoid changes and a severe inflammatory background. In addition, we should be alert to the possibility of LCH with malignant transformation if the histological morphology reveals tumor cell nucleoli and mitotic figures, especially with signs of atypical mitosis. The immunohistochemical marker CD56 may help differentiate between LCH and LCS when the morphology is difficult to determine. Molecular detection has shown that the mutation rate of *BRAF* in gastric LCH is up to 90.9%. This study has several limitations that must be acknowledged. First, the small sample size, which includes only 3 cases of gastric LCH, is insufficient to fully elucidate the clinicopathological and molecular features of the condition. Therefore, collecting more cases in the future is essential for a more comprehensive analysis. Secondly, due to the inherent limitations of retrospective studies, a prospective study design is recommended to provide a more robust evaluation of gastric LCH. Current data indicate a good prognosis for isolated gastric LCH in adults; however, to advance research on gastric LCH, it is essential to focus on expanding the sample size for more robust analysis, employing advanced molecular techniques to uncover genetic and molecular insights, conducting prospective studies for systematic data collection and accurate treatment assessments, and carrying out comprehensive clinical-pathological studies to deepen the understanding of disease pathology and improve diagnostics.

## Author contributions

**Formal analysis:** Ruinuan Wu.

**Investigation:** Ruinuan Wu.

**Methodology:** Ruinuan Wu, Xikang Wu.

**Project administration:** Ruinuan Wu, Huihui Gui.

**Supervision:** Ruinuan Wu, Yali Zhao, Xia Liu, Zhaohui Liu.

**Writing – original draft:** Ruinuan Wu.

**Resources:** Yali Zhao, Xikang Wu.

**Writing – review & editing:** Xia Liu, Zhaohui Liu.
